# Comparison of CDC Bottle Bioassay with WHO Standard Method for Assessment Susceptibility Level of Malaria Vector, *Anopheles stephensi* to Three Imagicides

**Published:** 2019-03-30

**Authors:** Hassan Vatandoost, Mohammad Reza Abai, Morteza Akbari, Ahmad Raeisi, Hemn Yousefi, Soraya Sheikhi, Akbar Bagheri

**Affiliations:** 1 Department of Medical Entomology and Vector Control, School of Public Health and National Institute of Health Research, Tehran University of Medical Sciences, Tehran, Iran; 2 Department of Chemical Pollutants and Pesticides, Institute for Environmental Research, Tehran University of Medical Sciences, Tehran, Iran; 3 Department of Malaria, Center for Disease Control (CDC), Ministry of Health and Medical Education, Tehran, Iran

**Keywords:** Susceptibility, Insecticide, WHO bioassay, CDC bioassay, *Anopheles stephensi*

## Abstract

**Background:**

The detection of insecticide resistance in natural populations of *Anopheles* vectors is absolutely necessary for malaria control. CDC bottle bioassay as a new tools has been employed for detecting the insecticide resistance. For a limit number of mosquito vectors, diagnostic doses and diagnostic times for some insecticides have already been determined using this new assay. For the first time in the area, susceptibility levels of *Anopheles stephensi* was done with DDT, deltamethrin, and bendiocarb using CDC bottle bioassay and compared results with WHO standard test method.

**Methods:**

*Anopheles stephensi* were collected in larvae stage from the cisterns of drinking water in Chabahar port which considered as old malaria foci, Sistan and Baluchistan province. The field collected larvae were colonized at the insectary of School of Public Health (SPH), Tehran University of Medical Science. The susceptibility tests were carried out on sugar fed female mosquitoes aged 2–3 days, against DDT 4%, bendiocarb 1% and deltamethrin 0.05% using WHO and CDC susceptibility methods. The mortality and knockdown rates, as well as the parameters of regression analysis, including LT_50_ and LT_90_, was calculated separately for the WHO and CDC methods.

**Results:**

The 24h mortality rates of *An. stephensi* were 28.6% and 25.6% for DDT, 60.8% and 64.6% for bendiocarb and 100% for deltamethrin using both WHO and CDC assay at 30 and 60min respectively. The 50% lethal times (LT_50_) were estimated 44.9 and 66.2min, 38.9 and 81.8min and 0.7 and 15.0min respectively using both WHO and CDC susceptibility tests.

**Conclusion:**

The similar results of susceptibility levels were shown for DDT, bendiocarb and deltamethrin. The lethal times (LT_50_) showed significant difference using both WHO and CDC bioassay methods.

## Introduction

Malaria and other mosquito-borne diseases are the major problems worldwide. Currently, there are proven and effective tools to combat against vectors (1).

More than 80% of malaria cases in the country are reported from three provinces southern and southeastern areas of the country (2). The most routes of malaria cases are immigratio from eastern borders (3) which affected by extensive population movement across the border with Pakistan, There are several works on different aspects of malaria in the country which is useful for decision making (4). Among of *Anopheles* mosquitoes, *Anopheles stephensi* Liston (Diptera: Culicidae) for both *Plasmodium falciparum* and *P. vivax* with the geographical range from the Middle East to India and China and southern slope of areas located in Zagros mountain chains (5). Recent studies in Iran have been revealed the presence of 31 *Anopheles* species including genotypes and sibling species, eight of them involved in malaria transmission in Iran (6). Vector control as the main measures used to reduce malaria transmission at the community level and considered as the most effective measure for eradicating malaria. It has been proven to be the only measure that can reduce malaria transmission from very high to a low level (7). Resistance to insecticides among the mosquito vectors is the most important growing concerns in many countries and bioassays allow for the detection and characterization of insecticide resistance in a vector population. World Health Organization (WHO) susceptibility test is the main assay for assessing the susceptibility /resistance status among mosquito vectors. CDC bottle bioassay is a new tool for assessing resistance to insecticides in the world (8). The goal of CDC bioassay is to measure the mortality rate of members of a population at a given dose of the insecticide. In order to know the advantages, disadvantages and features, the WHO test compared with the CDC bottle bioassay using three insecticides against *An. stephensi*.

The resistance parameters of this research allowed comparing data across other countries. To determine the diagnostic dose and the diagnostic time for use in the CDC bottle bioassay, the assay was calibrated.

## Materials and Methods

### Mosquito tested

The larvae of *An. stephensi* were collected from the artificial ponds in the urban area of Chabahar Port, Sistan and Baluchistan Province and colonized at the insectary of School of Public Health (SPH), Tehran University of Medical Science (TUMS). The rearing condition was 29±1 and 60–65% relative humidity with 12L: 12D photoperiod cycle. Larvae were fed with fish flakes and adults were fed a sugar water solution consisting of 10% sucrose. For mosquito mass colonization, female mosquitoes were fed on guinea pin hold in a restrainer. Females 3 to 4 days post emergence were used in all experiments.

### Insecticide materials

The choice of deltamethrin and bendiocarb were based on conventional use in residual spraying in malaria foci and DDT selected as indicator insecticide for revealing of resistance. The insecticide impregnated papers were purchased with WHO representative in Penang, Malaysia. The technical active ingredient of DDT, bendiocarb and deltamethrin were provided from Ecotoxicology Laboratory, School of Public Health, Tehran University of Medical Sciences.

### WHO’s susceptibility test protocol

In each replicate, 20 to 25 sugar-fed female mosquitoes aged 2–5 days gently released into the holding tubes with green dots (4–5 as treated group and 1 as control one) lined inside each tube with untreated papers following the WHO’s protocol (9). After resting the mosquitoes 15 minutes at the vertical position, they gently blown into the exposure tubes with red dots lined inside the tubes with WHO’s insecticide impregnated papers including DDT 4%, bendiocarb 1% and deltamethrin 0.05%. The mortality rate of *An. stephensi* was assessed at logarithmic trend around the diagnostic time. After each exposure time, the mosquitoes were transferred back into the holding tubes marked with green dots and provided the cotton pad moistened with the 10% sucrose solution at top of the nets. The knockdown and mortality rates were recorded after 60 minutes and 24 hours respectively. The environmental condition of test room was 28±1 °C and 60–65% relative humidity.

### CDC bottle test protocol

The CDC bottle test was conducted as a new surveillance tool for detecting the insecticide resistance among malaria vector at the given diagnostic doses and the diagnostic exposure times.

The stock solutions were prepared by adding 10 and 1.25μl of technical grade of DDT, deltamethrin and bendiocarb per 100ml pure ethanol. The inside surfaces of 250ml glass bottles were coated with 1ml of stock solution and control bottles were coated only with pure alcohol according to the CDC protocol. For each concentration five bottles were coated. The diagnostic doses of DDT, deltamethrin and bendiocarb for *Anopheles* species are respectively standardized as 100, 12.5 and 12.5μg/bottle (8). The coated bottles were placed in incubator equipped to ventilator and set temperature at 35 °C to allow to evaporate the solvent overnight. After the alcohol was fully evaporated, 15–25 mosquitoes aged 3–4 days fed with 10% sucrose solution were released to each bottle using a hand aspirator. The exposure time was set to 30min and for possible estimating of median lethal time (LT_50_), different exposure times 0.5, 1.0, 1.9, 3.8, 7.5, 15, 30, 45, 60, 75, 90, 105 and 120min were used. The mosquitoes were removed from the bottles and sorted into “alive” and “knocked-down” groups. Mosquito groups were kept in separate paper cups with 10% sucrose solution under insectary conditions. After 24h, they were scored as alive or dead in order to determine delayed mortality. At least five replicates were performed for each exposure time.

### Statistical analysis and data interpretation

The data interpretation for WHO susceptibility tests for susceptible/resistance status of *Anopheles* species were adopted according to the latest related criteria (1) as follows:

1. **Susceptible status:** When 98–100% mortality rate resulted at the recommended diagnostic dose and time.
2. **Tolerance status:** When, 90–97% mortality rate resulted at the recommended diagnostic dose and time which suggests the possibility of resistance and needs to be confirmed.
3. **Resistance status:** When less than 90% mortality rate resulted at the recommended diagnostic dose and time.

The data interpretation of the CDC bottle test was adopted according to the related protocol. The susceptibility thresholds was considered at the diagnostic time of 30 minutes for all insecticide tested at the recommended diagnostic doses (8):

1. **Susceptible status:** The tested mosquitoes with insecticide-coated bottles are considered susceptible when died before the diagnostic time.
2. **Resistance status:** If tested mosquitoes survive beyond diagnostic time, these survivors represent a proportion of the population with some degree of resistance. The susceptibility tests can be carried out beyond the diagnostic time to evaluate the intensity of resistance.

The data from the CDC bottle bioassay using test mosquitoes need to be compared with data from susceptible mosquitoes or from a population that will serve as baseline. Resistance thresholds for each insecticide can be determined by calibrating the CDC bottle bioassay.

The bioassay data were analyzed using the probit program and the values for the lethal times 50% and 90% mortality (LT_50_, LT_90_) and 95% confidence interval were estimated.

The Abbott’s formula was not applied to the mortality rate in treated and control group were less than 5% in all experiments (10). The regression line of the tested insecticides were plotted using Microsoft Excel (ver. 2013).

## Results

### Susceptibility to DDT

The 24h mortality rate of *An. stephensi* was 28.6% and 25.6 % exposed respectively 30min and 60min to DDT using WHO and CDC assays (Table 1). The logarithmic exposure times of DDT were ranged from 15 to 120min for CDC bottle test and from 30 to 240 minutes for WHO test. The 50% lethal times (LT_50_) were estimated 44.9min and 66.2min respectively. The DDT regression equations of the mortality *An. stephensi* were set as Y= −6.4192+3.5250X and Y= −4.9652+3.0045X with WHO and CDC assays (Table 2).

**Table 1.** Susceptibility data recorded according to both WHO and CDC methods against *An. stephensi*

**Insecticides**	**Susceptibility method**	**Range of exposure time (min)**	**Total tested**	**Range of mortality rate (%) at logarithmic exposure time**	**Mortality rate (%) after 24h maintenance**	**Resistance status**
**DDT**	WHO	30–240	371	18.6–100	28.6 (60min)	R*
	CDC	15–120	1107	13.9–100	25.6 (30min)	R
**Bendiocarb**	WHO	7.5–60	369	13.3–97.8	60.8 (60min)	R
	CDC	15–120	389	57.3–100	64.6 (30min)	R
**Deltamethrin**	WHO	0.47–120	380	28.3–100	100 (60min)	S**
	CDC	15–120	1080	100–100	100 (30min)	S

* Resistance

** Susceptible

**Table 2.** Probit regression line parameters of *Anopheles stephensi* exposed to different insecticides with WHO method

**Insecticide**	**Susceptibility assay**	**A**	**B±SE**	**LT_50_, 95% C.I. (Min)**	**LT_90_, 95% C.I. (Min)**	**X^2^ (df)**	**P value**
**DDT 4%**	WHO	−6.4192	3.5250±0.298	59.3593	131.8797	15.265 (2)	0.01
				**66.2271**	**152.9699**		
				73.6338	185.3884		
**DDT 1%**	CDC	−4.9652	3.0045±0.151	42.0031	107.3886	81.874 (2)	0.01
				**44.9348**	**119.9894**		
				48.0928	136.7425		
**Bendiocarb 1%**	WHO	−4.6479	2.9241±0.262	34.4114	88.9256	9.045 (2)	0.05
				**38.8617**	**106.6132**		
				43.7645	135.9259		
**Bendiocarb 1%**	CDC	−4.4782	2.3413±0.254	70.1951	209.6077	21.696 (2)	0.05
				**81.7899**	**288.4610**		
				98.9898	464.5222		
**Deltamethrin 0.05%**	WHO	0.3371	2.7535±0.249	0.651	1.8701	4.545 (2)	0.05
				**0.7543**	**2.2029**		
				0.8571	2.7275		
**Deltamethrin 1%**	CDC	−3.4038	2.8646±0.346	12.3959	36.7971	46.788 (2)	0.05
				**15.4251**	**43.2137**		
				18.0780	54.0015		

### Susceptibility to bendiocarb

The 24h mortality rates of *An. stephensi* were 60.8% and 64.6% exposed 30min and 60 min to bendiocarb using WHO and CDC assays (Table 1).

The exposure times of bendiocarb were ranged from 7.5 to 60min for WHO tests and from 15 to 120 minutes for CDC bottle tests. The 50% lethal times (LT_50_) were 38.9min and 81.8min respectively. The regression equations of the mortality *An. stephensi* were set as Y= −4.6479+2.9241X and Y= −4.4782+2.3413X with WHO and CDC assays (Fig. 2).

**Fig. 1.**
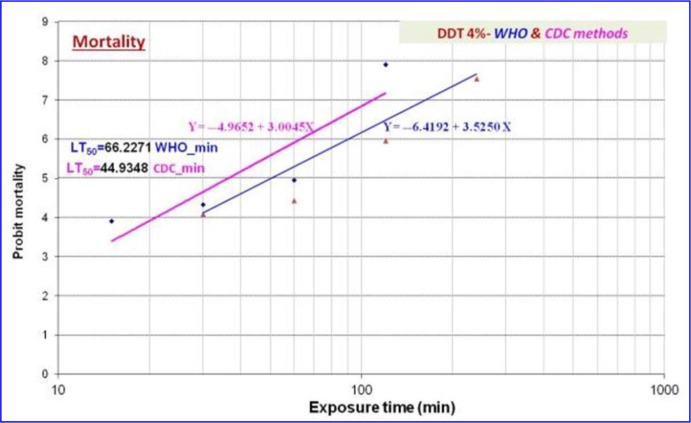
Mortality rate and regression analysis of bioassays of *Anopheles stephensi* (Chabahar strain) exposed to DDT using WHO and CDC methods

**Fig. 2.**
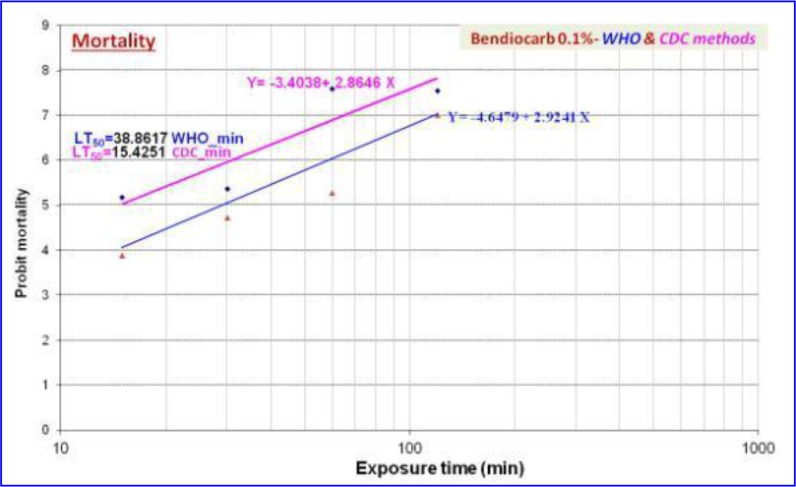
Mortality rate and regression analysis of bioassays of *Anopheles stephensi* (Chabahar strain) exposed to bendiocarb using WHO and CDC methods, 2015–2016

### Susceptibility to deltamethrin

The 24h mortality rates of *An. stephensi* were 100% exposed 30min and 60min to deltamethrin using WHO and CDC assays (Table 1).

**Fig. 3.**
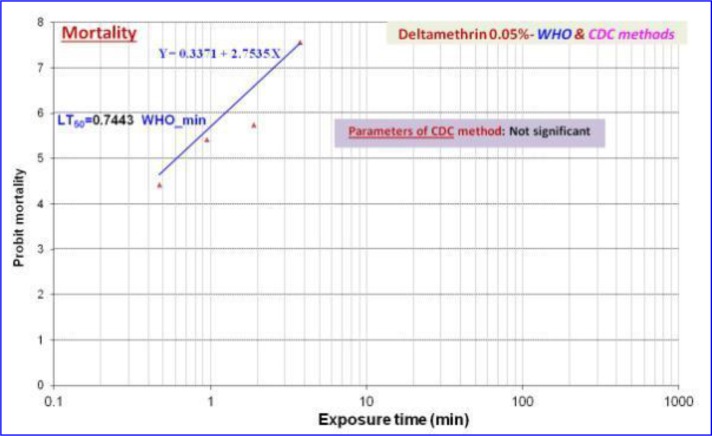
Mortality rate and regression analysis of bioassays of *Anopheles stephensi* (Chabahar strain) exposed to deltamethrin using WHO and CDC methods, 2015–2016

The exposure times of deltamethrin were ranged from 0.5 to 120min for WHO tests and from 15 to 120 minutes for CDC bottle tests. The 50% lethal times (LT_50_) were 0.8min and 15.4min respectively. The regression equations of the mortality *An. stephensi* were set as Y= 0.3371+2.7535X and Y= −3.4038+2.8646X with WHO and CDC assays (Fig. 3).

## Discussion

The WHO standard method is a major and widely used method in the country, but the CDC bottle bioassay method was used for the first time in order to compare the resistance levels. Information about the susceptibility of the vectors to insecticide is essential for chemical interventions, so routine determining the susceptibility levels of malaria vector is an integral part of vector control program. In this study, two important methods were used to perform susceptibility levels on malaria vector, *An. stephensi* that has been colonized at the insectary of SPH, TUMS which the first generation transported from Chabahar district have been showed resistance or tolerance to several types of insecticides including organochlorine, pyrethroid and carbamate (11). Approximately in all previously studies on susceptibility status of *An. stephensi* in Iran, resistance to DDT have been reported, although tolerance to DDT only reported from southeastern part of the country (12).

This is the first study for using CDC bottle test for detecting insecticide resistance in the mosquito vectors in Iran.

There are some published studies on using CDC bottle test for detecting DDT resistance on disease vectors with 30min exposure time including *An. gambiae* in Madagascar with 99 % mortality rate (13), same species in Nigeria with 25% mortality (14, 15), *An. gambiae* in Zambia with 90% (16) and on *An*. *nuneztovari* in Columbia with 85% mortality (17). Our finding also showed highly resistance of *An. stephensi* to DDT with similar values (25.6% and 28.6%) for mortality rate using both test methods. This is the second evidence of similarity of the results using both CDC and WHO bioassay method for revealing of susceptibility status compared the first one (22). The CDC bioassay conducted in the Macha, Zambia, the DDT at 300 μg/bottle, with delayed mortality 13–69% (18).

The CDC bioassay method was also applied to reveal the resistance status of malaria vectors to bendiocarb, with 30min exposure time including *An. gambiae* in the Benin with 78.9–100% mortality depending to different tested localities (19, 20), *An. gambiae* in northern Benin with 100% mortality both in the field (16) and laboratory condition (18). The CDC bottle assay test showed that *An. gambiae* s.l. was fully susceptible to bendiocarb in Nigeria (15) and in almost all of the 18 sampled districts in Ethiopia except in Omonada, which 25% mortality was recorded (14). Regarding *An. gambiae* from Tanguieta site, a mortality of 78.9% was recorded after exposure to CDC bottles treated with bendiocarb showing an indication of resistance of this population to this insecticide (20).

In this study, *An. stephensi* showed 57.3– 100% mortality (Table 1). Regarding *Anopheles gambiae* s.l. population from the Tanguieta, Benin, resistant to bendiocarb was reported according to both WHO and CDC methods with mortality rates of 56.14% and 78.94% respectively (16). With WHO tube test, the exposure of *An. stephensi* to the bendiocarb 0.1% showed 13.3–97.8% in this study, and 71.4% on *An. stephensi* in other study carried out in the Nikshahr, southeastern Iran (23).

Due to the extensive use of pyrethroids both for indoor residual spraying and impregnating bed nets and some reports for resistance occurrence among the *Anopheles* vectors (23–24), the comparative study using WHO and CDC were carried out for revealing the resistance level in the world. This study revealed the susceptibility of *An. stephensi* to the deltamethrin (Table 1, Fig. 3). Gorouhi et al. in 2015 indicated that field strain *An. stephensi* is the resistant candidate to deltamethrin (25).

A bioassays conducted in north and south Benin, 100% mortality of *An. gambiae* was also shown to deltamethrin with CDC assay at 12.5μg/bottle and 30min exposure time (21) as well as with deltamethrin 0.05% impregnated paper (22). The CDC insecticide susceptibility test was used on *An. funestus* and *An. Mascarensis* in three districts including Farafangana, Fenerive Est and Vavatenina in the Zambia and revealed the susceptibility of these species to the deltamethrin (13) and the high resistance of An. gambiae was revealed 0–90% mortality in Nigeria (15). In other study in Dangbo district, West Africa, the resistance of *An. gambiae* to deltamethrin was indicated both with CDC and WHO bioassay methods respectively with 73.8% and 50.8% mortality (27). The increasing of kdr allelic frequency correlated with the CDC bioassay data on Malanville and Sure-Lere population *An. gambiae* (27, 16) as well as the biochemical assays were implicated the mono-oxygenase enzymes as mechanisms of pyrethroid resistance in *An. gambiae* from Misserete, West Africa (28, 29). A diagnostic dose of 10μg a.i./bottle was identified as the most sensitive discriminating dose for characterizing resistance in *An. darlingi* and *Ae. aegypti* and both the bottle assay and the WHO assay were equally able to differentiate deltamethrin-resistant and susceptible *An. albimanus* populations (30).

There are some published studies on using CDC bottle assay for detecting pyrethroids resistance of malaria vectors with 3min exposure time including *An. gambiae* in Madagascar with 99% mortality rate (13), *An. gambiae* in Nigeria with 25% mortality (14, 15), *An. gambiae* in Zambia with 90% (16) and on *An*. *nuneztovari* in Columbia with 85% mortality (17).

The current study emphasizes that the results of both WHO and CDC bioassays were similar. Another recent study was also emphasizes that both WHO and CDC bioassays give similar results with regard to mosquito susceptibility to deltamethrin and bendiocarb insecticides (21). The efficacy Wheaton coated bottle with deltamethrin could be used at least three times during four consecutive days in laboratory conditions (22).

WHO method requires more mosquitoes than CDC method, the comparison between the results of both methods is clear. When the WHO susceptibility kit is not readily available, bottle bioassays can be used to determine insecticide resistance status of mosquito populations. WHO bioassays utilize cylinder plastic tubes whereas CDC bottles bioassays use 250 ml Wheaton bottles which are made of glass. World Health organization (WHO) papers do not need to be treated by oneself before their utilization because they are ordered in the impregnated form. Conversely, CDC bottles need to be coated with insecticide by oneself before each bioassay. In fact, the shelf life and reuse of prepared bottles are still not well documented or studied in laboratory conditions (21). However, in field conditions, the studies of Perea et al. showed that bottles treated with 10μg ai deltamethrin per bottle could be stored for at least 14 days and reused on three occasions (27). The major advantages of the bottle assays are that any concentration of a custom insecticide (pure or formulated) may be evaluated and the technique is simple, rapid and economical. One of the disadvantages of who method is the transfer of mosquitoes to the tubes, which can damage the mosquitoes and cause disturbances in the test results so requires care during WHO susceptibility tests. This problem is partly resolved in the CDC method because CDC bottles bioassays do not need mosquitoes to be transferred from the exposure bottle. In WHO susceptibility tests mosquitoes must remain in recovery period (stable conditions of temperature and relative humidity) during the 24 hours after exposure to insecticide impregnated paper. The environmental conditions that mosquitoes have in recovery period can affect the test results, which is one of the disadvantages of this method. In CDC bottle bioassays method, this problem has been solved. The CDC bottles need to be clean, dry and coated with insecticide by oneself before each bioassay that takes a long time. If the bottles are contaminated before the coating, there is an error in the test results and this is one of the disadvantages of CDC bottle bioassays method. The assessment of the diagnostic dosage and time for each insecticide used against malaria vectors, in each region and for each of the main vector species is absolutely necessary. According to the CDC method, any concentration of any insecticide (pure or formulated) may be evaluated.

WHO insecticide susceptibility test is the most common method for assessing resistance status in Iran. In this study, for the first time in the country, CDC bottle bioassay method has been used to evaluate the level of mosquito susceptibility to the conventional insecticides, and additional supplementary studies are required.

## Conclusion

The current study emphasizes that the results of both WHO and CDC bioassays were similar with the studied insecticides with no significant difference in the related values. The CDC bottle bioassay may be applied as part of a broader insecticide resistance monitoring program especially combined with results of bioassays using synergists and those of biochemical and molecular assays. Also the CDC bioassay have been used for determining of biochemical mechanisms that involve the detoxifying enzymes.
